# New polymorph of tofacitinib citrate and in vitro equivalence study of its sustained-release tablets

**DOI:** 10.1371/journal.pone.0332664

**Published:** 2025-10-14

**Authors:** Yan Wang, Shuai Wen, Yao Zhao, Lijuan Gui, Yi Mou, Dongkai Wang

**Affiliations:** 1 Department of Pharmaceutics, School of Pharmacy, Shenyang Pharmaceutical University, Shenyang, People's Republic of China; 2 School of Pharmacy, Taizhou University, Taizhou, People's Republic of China; National University of Rosario, ARGENTINA

## Abstract

Tofacitinib citrate (TOC) is clinically used primarily for the treatment of moderate to severe rheumatoid arthritis in patients who are intolerant or inadequately responsive to methotrexate. In this study, a new polymorph of TOC (form II) was prepared using solvent crystallization. The currently marketed form I and the self-prepared form II were characterized by XRD, DSC, FT-IR, and SEM, confirming that form II is a new polymorphic form of TOC. This new polymorph was then used in the development of TOC sustained-release tablets. In the formulation design, mannitol with low hygroscopicity was used to replace sorbitol. By adding a certain amount of microcrystalline cellulose to the tablet core and maintaining the mannitol content between 60 ~ 70% of the total core weight, an in vitro dissolution behavior similar to that of the reference formulation (*f*_2_ > 50) was achieved. This indicates that replacing sorbitol with mannitol as the osmotic agent in the osmotic pump tablet core is a practical and effective method. Additionally, key preparation processes for the TOC sustained-release tablets were investigated. The results suggest that the coating weight gain of the self-prepared sustained-release tablets should be controlled between 6% and 7%, and the suitable range for laser-drilled orifice diameter is 0.55 ~ 0.90 mm.

## 1. Introduction

**Fig 1:**
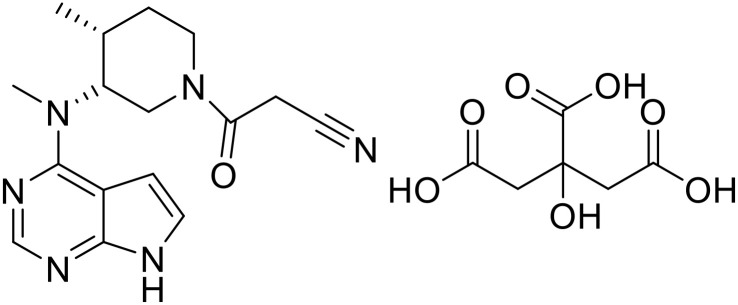
The structure of TOC.

Tofacitinib citrate (TOC) is a novel oral small-molecule JAK3 inhibitor and is clinically used as the first-line treatment for moderate to severe rheumatoid arthritis in patients who are intolerant or inadequately responsive to methotrexate. Its molecular structure is shown in Fig 1, and it is classified as a BCS III drug [1]. Pfizer has developed and marketed both immediate-release and sustained-release tablets of tofacitinib citrate. The sustained-release tablets are sold under the brand name Xeljanz XR, with available strengths of 11 mg and 22 mg. The active pharmaceutical ingredient (API) used in these marketed products is the polymorph protected by Pfizer’s patent EP1451192 [2], We refer to this polymorph as form I。In the development of solid dosage forms, polymorphism is a common phenomenon in APIs. Different polymorphs of API may exhibit distinct physicochemical properties, such as melting point, solubility, dissolution rate, and density. When a compound exhibits polymorphism, the different crystalline forms of API can significantly impact the stability and bioavailability of the formulation [3]. In addition to form I, Pfizer’s patent WO2012/135338 discloses the preparation method for the non-crystalline form (amorphous) of the compound [4]. In recent years, there has been limited research on the polymorphism of TOC. Only Kaduk et al. have solved and refined the crystal structure published in patent CN1325498C using XRD data and further optimized it using density functional theory [5]。

Polymorph research of the API is a critical component of pre-formulation studies in the development of solid dosage forms, while formulation design is the core aspect of pharmaceutical research. Pfizer’s patent WO2014147526A1

discloses that the controlled drug release technology used in its sustained-release tablets is based on the single-chamber osmotic pump system [6], This drug release technology was first proposed by Waterman [7], it utilizes high-molecular-weight polymers, such as hydroxyethyl cellulose, as swelling agents in the tablet core; highly water-soluble low-molecular-weight substances, such as NaCl, sorbitol or mannitol, as osmotic agents in the core; and water-insoluble polymers, such as cellulose acetate or ethyl cellulose, as semipermeable membrane materials to coat the tablet core. Finally, a hole is created at one end of the tablet through laser or physical drilling. When water penetrates the semipermeable membrane and enters the tablet core, the osmotic agent rapidly dissolves, creating a high osmotic pressure. The high-molecular-weight polymer in the core continuously hydrates and swells, pushing the drug out through the release hole, thereby controlling the drug release rate. Xeljanz XR uses sorbitol as the osmotic agent to promote drug release, with its content ranging from 60% to 85%. However, sorbitol is a highly hygroscopic excipient, often causing sticking during the tableting process. This requires special control of environmental humidity during preparation, posing challenges for industrial-scale production. Additionally, since sorbitol is metabolized into fructose in the body, patients with hereditary fructose intolerance cannot take medications containing sorbitol. There are also reports in the literature that sorbitol has a nonspecific laxative effect, which can be achieved with a daily intake exceeding 50 g. Given that the sorbitol content in the original formulation is as high as approximately 76%, there is a potential risk of laxative side effects [8].

Mannitol is widely used as API for the treatment of elevated intracranial pressure, renal protection during cardiac, vascular, and kidney transplant surgeries, as well as for the management of rhabdomyolysis [9]. Mannitol itself is also a commonly used pharmaceutical excipient and is increasingly being utilized in solid oral dosage forms. Compared to sorbitol, mannitol exhibits low hygroscopicity, excellent compressibility, and good compatibility with most APIs. Importantly, it does not produce the fructose-related effects in patients that are associated with sorbitol, making it a more favorable alternative in formulations [8]. Literature reports indicate that when tablets are prepared using both sorbitol and mannitol, the drug dissolution rate is higher in tablets with sorbitol as the filler compared to those with mannitol. This suggests that for sustained- and controlled-release formulations designed to achieve slow drug release, mannitol is more effective in slowing down the drug release rate [10].

Among various excipients, mannitol and sorbitol are recognized as key excipients that significantly alter drug absorption through osmotic effects, particularly for low-permeability drugs classified under BCS III and IV. Due to their inherent osmotic properties, these excipients generate osmotic pressure in the body, which can accelerate intestinal transit, reduce drug absorption, and ultimately lower bioavailability [11,12]. Literature has confirmed the colligative properties of osmotically active excipients, meaning that the number of excipient molecules in a solution (rather than their concentration) determines the osmotic pressure exerted by the excipients in the body [13]. As an isomer of sorbitol, mannitol has a slightly higher isotonic equivalent value compared to sorbitol when paired with sodium chloride. The isotonic equivalent value (E) of mannitol is 0.18 per 1 g of the drug (compared to 0.16 for sorbitol). This indicates that in osmotic pump-type sustained-release tablets, using a smaller amount of mannitol as the osmotic agent instead of sorbitol can achieve the same osmotic pressure while reducing the number of osmotically active molecules entering the body. This, in turn, minimizes the impact on the absorption of BCS III drugs and improves their bioavailability. Furthermore, a literature review examined the usage levels of mannitol in commercially available drugs. A quantitative analysis of mannitol content in oral medications (16 active pharmaceutical ingredients, 132 products) marketed by 11 generic drug manufacturers in Japan revealed that the “actual” amount of mannitol used in formulations did not significantly affect oral drug absorption. This further supports the conclusion that mannitol is an excipient with a relatively minor impact on the absorption of low-permeability drugs when used as an osmotic agent [14].

In recent clinical treatments over the past five years, literature has reported that TOC can also be used to treat other immune-related conditions, such as organ transplantation, lupus, multiple sclerosis, psoriasis, and diabetes complications, among others. This demonstrates its broad clinical applications beyond its primary use [15–18]. Given this, tofacitinib citrate sustained-release tablets have also become a highly sought-after product among generic drug companies. Based on preliminary research, this study successfully prepared a new pharmaceutical polymorph of tofacitinib citrate, which we refer to as form II. This new polymorph was utilized in the development of TOC sustained-release tablets. During the optimization of the formulation and preparation process, mannitol was used to replace sorbitol as the osmotic agent in the tablet core. Key process parameters, such as coating weight gain and laser-drilled orifice diameter, were investigated to prepare sustained-release tablets with in vitro dissolution behavior similar to that of the reference listed drug.

## 2. Expermental section

### 2.1 Chemicals and materials

TOC (Wuhan Xinxin Jiali Biotechnology Co., Ltd.); TOC sustained-release tablets (Xeljanz XR, Pfizer, Batch No.: DD9555); D-Mannitol (Hebei Samus Pharmaceutical Co., Ltd.); Sorbitol (Shanghai Huyuan Pharmaceutical Co., Ltd.); Microcrystalline cellulose (MCC, Model: PH102, Asahi Kasei (China) Co., Ltd.); Hydroxyethyl cellulose (HEC, Model: 250M PHARM, Nanjing Xinding Pharmaceutical Technology Co., Ltd.); Copovidone (CP, Model: VA64, Shanghai Yunhong Chemical Excipients Technology Co., Ltd.); Magnesium stearate (MS, Meixinjia Zhongwei Pharmaceutical Co., Ltd.); Cellulose acetate complete coating premix (CACP, Model: 500F190007-CN, Shanghai Colorcon Coating Technology Co., Ltd.); Hydroxypropyl cellulose (HPC, Model: EF, Nanjing Xinding Pharmaceutical Technology Co., Ltd.); Film coating premix (FCP, Model: 03K140024-CN, Shanghai Colorcon Coating Technology Co., Ltd.); Common reagents such as acetone, sodium hydroxide, concentrated hydrochloric acid, glacial acetic acid, ethanol, acetonitrile, potassium dihydrogen phosphate, sodium dihydrogen phosphate, and disodium hydrogen phosphate are of analytical grade (Shanghai Titan Technology Co., Ltd.).

### 2.2 Preparation of TOC crystal form II

Using the solvent crystallization method [19], 100 g of TOC was weighed and dissolved in 2000 mL of a mixture of methanol, acetonitrile, and water (volume ratio 1:1:1). The solution was heated under reflux for 1 hour, then cooled to 20 °C. Subsequently, 2000 mL of a pre-cooled mixture of ethanol and acetone (volume ratio 1:3) at 10 °C was added dropwise to the solution at a rate of 1 mL/min. After crystals formed, the temperature was further reduced to 0 °C in an ice-water bath, and the mixture was stirred under these conditions for 3 hours. The solution was then allowed to stand for crystallization for 4 hours, followed by filtration. The filter cake was dried under reduced pressure at 40 °C for 10 hours, yielding a white crystalline powder.

### 2.3 Characterization of polymorphs in TOC

#### 2.3.1 X-ray diffraction (XRD).

To obtain the crystallinity of formⅠand formⅡ, X-ray diffraction was used (D2 Phaser X-ray Diffractometer, Bruker, Germany). The analytical angle intervals of 2θ was in the range of 5 ~ 40º, and the digitalization speed was 5º/min [20].

#### 2.3.2 Differential scanning calorimetry (DSC).

DSC instrumentation (DSC1 Synchronous Thermal Analyzer, Mettler Toledo, USA) was used for formⅠand formⅡperform differential scanning calorimetry. Individual samples of formⅠand formⅡwere weighed directly in the pierced DSC aluminum pan and then were scanned in the temperature range of 40 ~ 300 °C with a heating rate of 10 °C/min under an atmosphere of dry nitrogen [21].

#### 2.3.3 Fourier transformed infrared spectroscopy (FTIR).

FTIR spectra were recorded on an IR prestige spectrophotometer (Nicolet 10 Fourier Transform Infrared Spectrometer, Thermo Fisher, USA) in the range of 4000 ~ 400 cm − 1 and resolution of 4 cm − 1 [22].

#### 2.3.4 Scanning electron microscopy (SEM).

The morphological characterization of formⅠand formⅡwas performed using a scanning electron microscope (Sigma 300 Scanning Electron Microscope, ZEISS, Germany) at an acceleration voltage of 3 kV. The samples were mounted onto a silicon wafer and air-dried overnight [23].

#### 2.3.4 Determination of solubility.

According to the method reported in the literature [24], the content of TOC was determined by HPLC system (Ultimate 3000 HPLC, Thermo Fisher, USA). A WondaCract ODS-2 column (4.6 mm × 250 mm, 5 µm) was used as the stationary phase. The mobile phase consisted of a 10 mmol/L potassium dihydrogen phosphate solution (adjusted to pH 6.8 with 0.1 mol/L sodium hydroxide solution) and acetonitrile (v:v = 75:25). The flow rate was set at 1.0 mL/min, the detection wavelength was 289 nm, the column temperature was maintained at 30°C, and the injection volume was 10 µL. The solubility of two forms in different pH conditions was investigated (pH 1.0 HCl solution; pH 3.0 and 4.0 acetic acid solution; pH 6.8 phosphate buffer solution; pH 7.0 water and pH 8.0 phosphate buffer solution)2.4 Preparation of TOC sustained-release tablets.

#### 2.4.1 Preparation of tablet core.

According to the formulation design referenced from the original patent [6], mix TOC form II, mannitol, hydroxyethyl cellulose, copovidone, and microcrystalline cellulose in the prescribed proportions uniformly using a three-dimensional mixer (Turbula mixer, WAB, Switzerland). Then, add the prescribed amount of magnesium stearate and mix thoroughly. Using a 10.5 × 5.2 mm oval shallow concave punch (DP30A Single-Punch Tablet Press, Beijing Guoyao Longli Technology Co., Ltd., China), compress the mixture into tablets with a theoretical weight of 200 mg. Adjust the main compression force of the tablet press to 5 ~ 40 kN, ensuring tablet hardness ranges between 50 ~ 130 N. The weight variation should be within ±5%, and the friability loss should not exceed 1%.

#### 2.4.2 Tablet coating and laser drilling.

Weigh the prescribed amounts of acetone and purified water and place them into a solution preparation tank. After stirring evenly, add the prescribed amount of hydroxypropyl cellulose EF and stir for 30 minutes to ensure complete dissolution. Then, add the cellulose acetate complete coating premix and stir for 1 ~ 2 hours to prepare the coating solution. Turn on the inlet and exhaust air for preheating. Transfer the tablet cores prepared in section 2.3.1 into the coating pan (BG1–5 high-efficiency coating machine, Guangzhou Jiacheng Pharmaceutical Equipment Co., Ltd., China). Set the inlet air temperature to 30 ~ 60°C, pan rotation speed to 13–16 rpm, inlet air volume to 1000 ~ 1500 m³/h, and coating rotation speed to 15 ~ 30 rpm, while maintaining the tablet bed temperature at 13 ~ 25°C. After coating is completed, place the coated tablets in an oven set at 40 ~ 50°C and dry for 24 hours. Then, use a laser drilling machine (CER-D30M laser drilling machine, Wuhan Chris Photoelectric Technology Co., Ltd., China) to create a small hole at the top of the oval tablets.

### 2.5 Determination of dissolution rate for sustained-release tablets

According to method II (paddle method) in the General Rules of the 2020 Edition of the Pharmacopoeia of the People’s Republic of China [25], a sinker was installed in the dissolution apparatus (708–850DS automated dissolution tester, Agilent, USA). Using 900 mL of pH 6.8 phosphate buffer solution as the release medium, the test was conducted at a temperature of 37°C and a paddle speed of 50 rpm. Samples of 5 mL were taken at 1 h, 1.5 h, 2 h, 2.5 h, 3 h, 4 h, 6 h, and 8 h (with an equal volume of release medium replenished after each sampling). The samples were filtered through a 0.45 μm microporous membrane, and the content was determined using the HPLC method described in section2.3.4. The cumulative dissolution rate was calculated, and the similarity factor (*f*_2_) method was used to evaluate the fit between the self-prepared formulation and the reference formulation.


$$f_{\text{2}}=\text{5}\text{0}\times \left\{{\left\lceil \text{1}+\frac{\text{1}}{n}\sum (R_{i}-{T_{i})}^{\text{2}}\right\rceil }^{-\text{0}.\text{5}}\times \text{1}\text{0}\text{0}\right\}$$

### 2.6 Key formulation and preparation process investigation

#### 2.6.1 Investigation of types and ratios of osmotically active substances in the tablet core.

The types and ratio ranges of osmotically active substances in the tablet core are shown in Table 1. Following the method described in section 2.4, seven batches of TOC sustained-release tablets were prepared. Using the commercially available TOC sustained-release tablets (Xeljanz XR, Pfizer, Batch No: DD9555) as the reference formulation, the dissolution rates of both the self-prepared sustained release tablets and the reference formulation were determined according to section 2.5, and the dissolution curves were plotted.

**Table 1:** Osmotically active substances and ratio settings.

Composition of osmotically active substances		Sorbitol	Sorbitol+ Mannitol	Mannitol+ MCC(1)	Mannitol+ MCC(2)	Mannitol+ MCC(3)	Mannitol+ MCC(4)	Mannitol
Tablet Core	API	8.885%						
	Sorbitol	76.115%	58.165%	/	/	/	/	/
	Mannitol	/	17.950%	48.183%	60.125%	67.934%	69.967%	76.115%
	MCC	/	/	27.932%	15.990%	8.181%	6.148%	/
	HEC	8.000%						
	CP	6.000%						
	MS	1.000%						
Sustained- release coating layer	CACP	2.780%						
	HPC	2.220%						
	Acetone	88.000%						
	Purified Water	7.000%						

#### 2.6.2 Investigation of coating weight gain.

Based on the optimal tablet core formulation determined in section 2.6.1, four batches of TOC sustained-release tablets with coating weight gains of 5.1%, 6.2%, 7.1%, and 8.0% were prepared according to the method described in section 2.4. Dissolution curves were plotted using the same method as in section 2.6.1.

#### 2.6.3 Investigation of laser drilling aperture size.

Based on the optimal tablet core formulation and coating weight gain determined in sections 2.6.1 and 2.6.2, three batches of TOC sustained-release tablets with laser drilling sizes of 0.2 × 0.2 mm, 0.4 × 0.4 mm, and 0.7 × 0.7 mm were prepared according to the method described in section 2.4. Dissolution curves were plotted using the same method as in Section 2.6.1.

### 2.7 Quality testing of self-prepared TOC sustained-release tablets

#### 2.7.1 Determination of dissolution curves in four dissolution media.

Using the optimal tablet core formulation, coating weight gain, and laser drilling size determined in section 2.6, three batches of TOC sustained-release tablets were prepared according to the method described in section 2.4. Dissolution testing was conducted in four media: pH 6.8 phosphate buffer solution, pH 4.5 acetate buffer, pH 1.2 hydrochloric acid solution, and water, following the method outlined in section 2.5. Dissolution curves were plotted using the same method as in Section 2.6.1.

#### 2.7.2 Static release and osmotic pressure determination.

An osmometer (YP-STY1 Freezing Point Osmometer, Shandong Youyunpu Photoelectric Technology Co., Ltd., China) was used to measure the osmotic pressure of the solution released at various time points in the water medium as described in section 2.7.1. The weight of the tablets at each time point was also measured.

## 3. Results and discussion

### 3.1 Polymorphism of TOC

**Fig 2:**
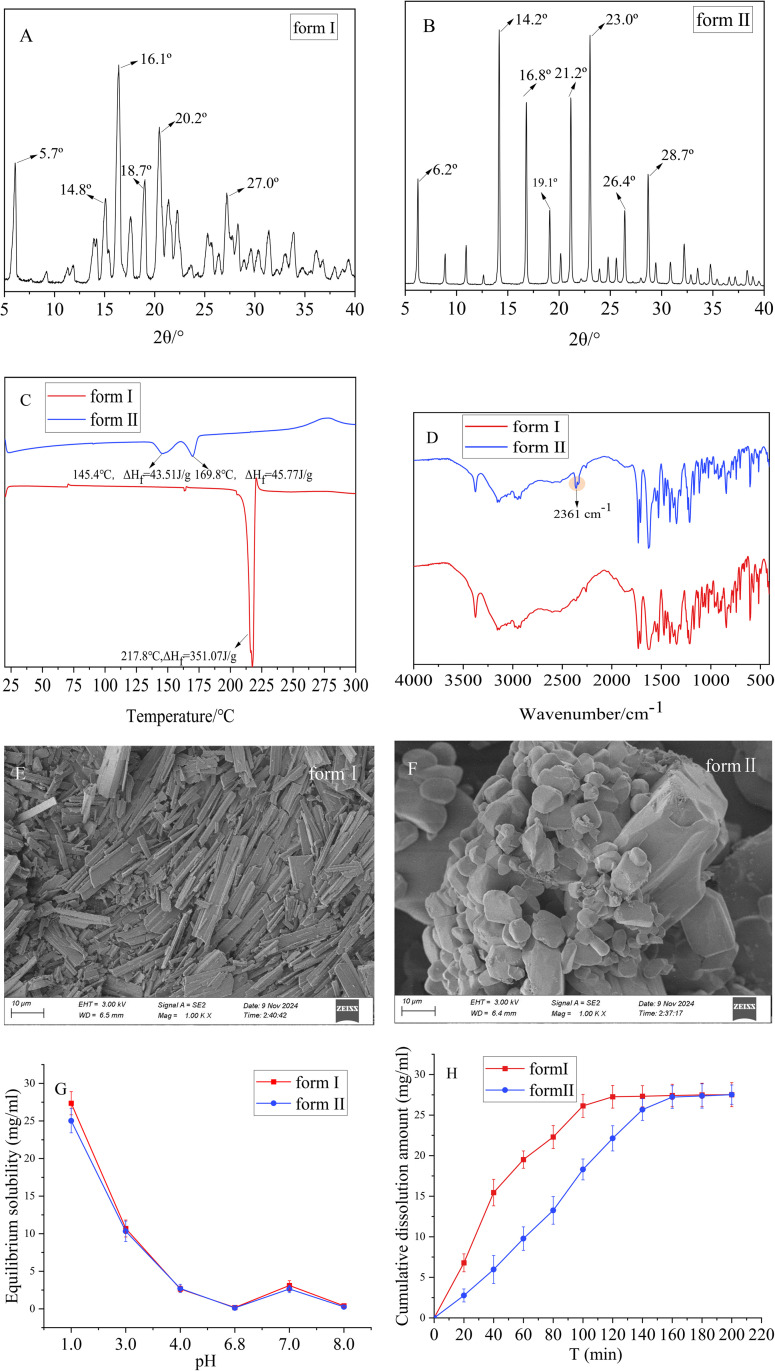
Characterization of different crystal types of TOC. (A-B) XRD of formⅠand formⅡ. (C) FTIR of formⅠand formⅡ.(D) DSC of formⅠand formⅡ. (E-F) SEM of formⅠand formⅡ. (G) Equilibrium solubility of formⅠand formⅡin dissolution media with different pH. (H) Cumulative dissolution amount of formⅠand formⅡ in pH1.0 HCl solution.

The XRD pattern of form I exhibits strong characteristic peaks (relative intensity greater than 30%) at 5.7°, 14.8°, 16.1°, 18.7°, 20.2°, and 27.0° expressed in 2θ diffraction angles, which is consistent with the original patent report [2]. The XRD pattern of form II exhibits strong characteristic peaks at 6.2°, 14.2°, 16.8°, 19.1°, 21.2°, 23.0°, 26.4°, and 28.7°, which are distinctly different from those of form I (Fig 2A and 2B). From the DSC curves, it can be observed that form I exhibits a distinct characteristic endothermic peak at 217.8 °C (ΔHf 351.07 J/g) while the original patent reports its characteristic endothermic peak is 203.16 °C [2]. The discrepancy in the melting endothermic peaks may be attributed to variations in detection conditions (such as heating rate), which can introduce certain deviations in experimental results. On the other hand, form II shows two endothermic peaks at 145.4 °C and 169.8 °C (Fig 2C) while ΔHf are 45.51 J/g and 45.77 J/g, which are markedly different from those of form I under the same experimental conditions. The FTIR spectra reveal that form I and form II exhibit similar characteristic absorption peaks in the functional group region (3500 ~ 2900 cm ⁻ ¹) and fingerprint region (2000 ~ 500 cm ⁻ ¹), which are associated with the pyrimidine-pyrrole ring in the molecular structure of TOC. However, form II exhibits a characteristic absorption peak at 2361 cm ⁻ ¹ (Fig 2D), the appearance of additional characteristic absorption peaks may be attributed to the differences in molecular arrangements between crystal forms [26]. SEM results (Fig 2E and 2F) show that form I exhibits a rod-like structure while form II consists of irregular granular particles with larger particle sizes. The particle size of API influences the dissolution behavior of formulation, larger particle sizes result in slower dissolution generally. The solubility determination experiment (Fig 2G and 2H) indicates that the overall trend of equilibrium solubility for form I and II is similar across varying pH conditions, both exhibiting significant pH dependence. The solubility peaks in a medium with a pH of 1.0 HCl solution. However, a notable difference in the dissolution rates of the two forms is observed in pH 1.0 HCl solution. The dissolution rate of form I is significantly higher than that of form II, reaching dissolution equilibrium within 120 minutes. In contrast, the dissolution rate curve of form II between 40 and 140 minutes approximates a straight line, indicating a constant-rate dissolution trend, with dissolution equilibrium achieved after 160 minutes. In summary, the combination of characterization techniques such as XRD, DSC, FTIR, and SEM demonstrates that crystal form II represents a novel polymorphic form, which can offer advantages in the development of TOC sustained-release tablets [27].

### 3.2 The key formulation and preparation process of TOC sustained-release tablets

**Fig 3:**
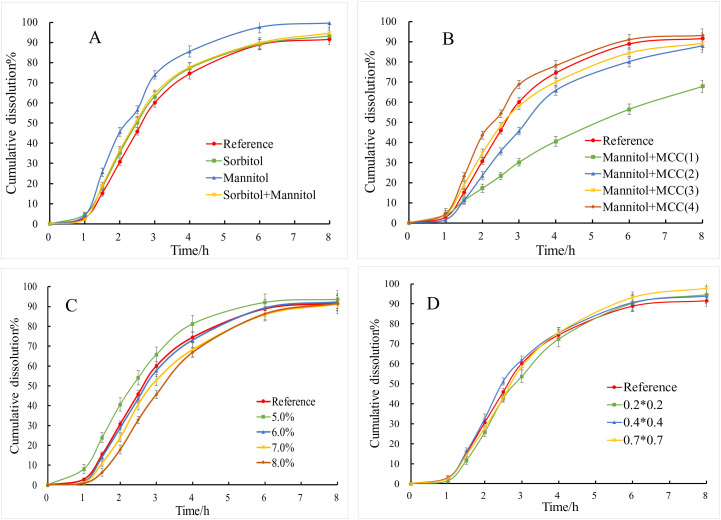
Dissolution curves of TOC sustained-release tablets. (A) Different osmotic pressure enhancers. (B) Different combinations of mannitol and MCC. (C) Different weight gain of slow-release coating. (D) Different aperture size, n = 6, ‾*x* ± *s*.

When sorbitol (w/w 76.115%) is selected as the sole osmotic pressure-active substance, the *f*_2_ value is 73.4. However, when a portion of the sorbitol is replaced with mannitol (sorbitol: 58.165%, mannitol: 17.950%), the dissolution rate shows a slight increase, but the *f*_2_ value remains at 68.0. Upon complete substitution with mannitol, the dissolution rate significantly improves, and the *f*_2_ value drops to 46.9 (<50), indicating that the sustained-release layer shell ruptures after 8 hours. This phenomenon occurs because the osmotic equivalent of mannitol is greater than that of sorbitol, meaning that at the same concentration, the osmotic pressure of mannitol exceeds that of sorbitol, leading to the rupture of the sustained-release layer coating [28] (Fig 3A). To reduce the amount of mannitol in the tablet core and simultaneously enhance the powder flowability for direct compression, microcrystalline cellulose is incorporated into the tablet core as a diluent [29], The results indicate that the dissolution rate decreases as the amount of mannitol is reduced. Specifically, when the mannitol content is 48.183% (w/w), the dissolution rate is significantly slower, yielding *f*_2_ value of 29.9. In contrast, when the mannitol content is between 60% ~ 70%, the *f*_2_ values consistently exceed 50. Notably, at a mannitol content of 67.934% (w/w) combined with 8.181% microcrystalline cellulose (MC), the formulation demonstrates the highest degree of similarity to the reference drug product, achieving an *f*_2_ value of 71.3 (Fig 3B). Hence, it is concluded that the optimal formulation for the TOC sustained-release tablets comprises 67.934% (w/w) mannitol as the osmotic pressure active agent in the tablet core, supplemented by 8.181% (w/w) MC as a diluent.

The thickness of the coating membrane in sustained-release tablets has a significant impact on drug release [30–32], a thicker coating membrane impedes the penetration of moisture into the tablet core, resulting in a slower dissolution rate. Conversely, a thinner coating membrane allows moisture to enter more rapidly, leading to an accelerated dissolution rate and potentially causing the membrane to rupture [33]. The results indicate that when the coating weight gain ranges from 5% to 8%, all *f*_2_ values exceed 50. The highest degree of similarity to the reference drug product is observed when the coating weight gain is between 6% ~ 7%. Consequently, it is concluded that the optimal coating weight gain for TOC sustained-release tablets is 6% to 7% (Fig 3C).

**Fig 4:**
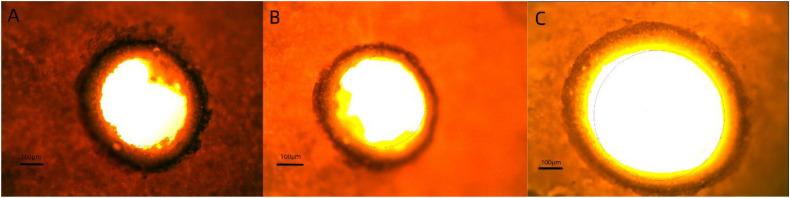
Pictures of the hole diameters after punching with different laser sizes. (A) 0.2*0.2 mm. (B) 0.4*0.4 mm. (C) 0.7*0.7 mm.

Laser drilling is the core technology of osmotic pump-controlled release tablets [34–37]. When water permeates the semipermeable membrane of the controlled-release coating, the osmotically active substances within the tablet core dissolve, resulting in the generation of osmotic pressure. This pressure facilitates the continuous release of API and the tablet core contents through the orifice, thereby achieving controlled drug release. Furthermore, the orifice size significantly influences the rate of drug release [38–40]. When the laser-drilled orifice size is 0.2 × 0.2 mm, although the *f*_2_ value remains greater than 50, the drug exhibits shell rupture during the release process due to the excessively small orifice size. In contrast, when the orifice sizes are 0.4 × 0.4 mm and 0.7 × 0.7 mm, the *f*_2_ values are 78.2 and 78.3 respectively, indicating good similarity with the reference formulation. This suggests that within a certain range, the orifice size of the controlled-release tablet does not significantly impact drug release (Fig 3D). After laser drilling, the specific dimensions of the orifices in the controlled-release tablets were measured using electron microscopy. The sizes of the orifices ranged from 0.45 to 0.49 mm for tablets drilled with a 0.2 × 0.2 mm laser, from 0.58 to 0.66 mm for those drilled with a 0.4 × 0.4 mm laser, and from 0.86 to 0.93 mm for those drilled with a 0.7 × 0.7 mm laser. Based on the results of the dissolution curve, the TOC sustained-release tablets exhibited an optimal release rate when the laser-drilled orifice size was within the range of 0.55 to 0.90 mm (Fig 4).

### 3.3 Quality evaluation of home-made TOC sustained-release tablets

**Fig 5:**
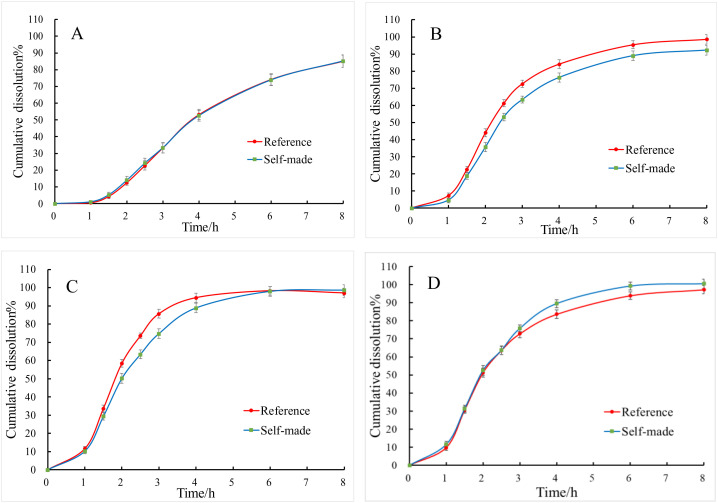
Dissolution curves in different dissolution medium. (A) pH 6.8 phosphate buffer. (B) pH 4.5 acetate buffer. (C) pH1.2 HCl. (D) water, n = 6, ‾*x* ± *s*).

In the evaluation of solid generic drugs, phosphate buffer solution at pH 6.8, acetate buffer at pH 4.5, hydrochloric acid solution at pH 1.2, and water were selected as the in vitro drug release media. The *f*_2_ value was calculated to predict bioequivalence [41]. The self-made sustained-release tablets, utilizing TOC form II as the active pharmaceutical ingredient, demonstrated *f*_2_ values of 71, 56, 56, and 72 across the four media, all exceeding 50. This suggests that in vitro dissolution behavior of the self-made formulation closely resembles that of the reference preparation (Fig 5).

**Fig 6:**
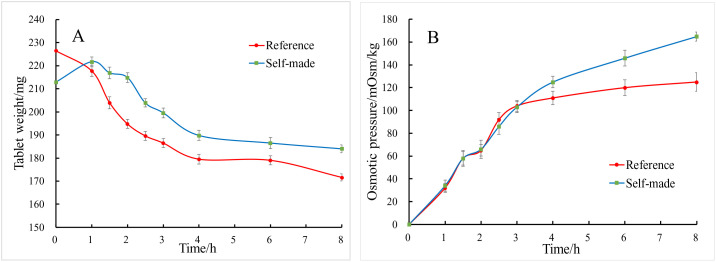
Static release and osmolality test. (A) static release test. (B) Osmolality test.

The static release experiment entails immersing the drug in an aqueous solution and monitoring the weight change of the tablet as the drug is released through small pores. In the development of osmotic pump-based controlled and sustained-release formulations, comparing the static release curves provides a more accurate prediction of the bioequivalence between the self-made preparation and the original formulation [42]. Moreover, osmotic pressure is a crucial parameter for evaluating the in vitro release of drugs from osmotic pump-controlled release formulations [43–45], when the osmotic pressure of a self-made formulation is similar to that of the original formulation, their in vivo bioavailability can also be comparable. The results indicate that both the self-made formulation and the reference formulation generally maintain consistent weights following static release in water. However, the weight of the self-made formulation exhibits a slight increase after one hour of release (Fig 6A). This phenomenon can be attributed to the incorporation of microcrystalline cellulose into the core of the self-made tablets as a diluent, which possesses a mild water-absorbing effect, thus contributing to the observed weight increase。Initially, the osmotic pressure of the self-made formulation was comparable to that of the reference formulation. However, after three hours, the osmotic pressure of the self-made formulation exceeded that of the reference formulation (Fig 6B). This increase can be attributed to the majority of the osmotic agent, mannitol, being released into the solution during the later stages. Given that the osmotic pressure of mannitol is greater than that of sorbitol, the osmotic pressure of the self-made formulation subsequently increased [46].

## 4 Conclusion

The study of polymorphs in APIs is of considerable significance for both new drug development and generic drug formulation. In this research, a novel polymorphic form (form II) of TOC was successfully prepared through solvent crystallization, which may represent a breakthrough relative to the original patent. In the subsequent formulation development, form II was utilized as the API, and mannitol, known for its lower hygroscopicity, was substituted for sorbitol as the osmotic agent in the tablet core. It was observed that the exclusive use of mannitol resulted in excessive osmotic pressure and rapid dissolution. However, the incorporation of a specific amount of microcrystalline cellulose yielded an in vitro dissolution profile comparable to that of the reference formulation. This indicates that replacing sorbitol with mannitol as the osmotic agent in the osmotic pump tablet core is both practical and effective. The results of this study suggest that the proportion of mannitol should be controlled within 60 ~ 70% of the total core weight. Additionally, the weight gain of the coating on the self-sustained release tablets should be maintained between 6% and 7%, and the diameter of the laser-drilled orifice should fall within the range of 0.55 ~ 0.90 mm. The findings of this research, through the development of a new drug polymorph and the refinement of formulation design and preparation processes, provide valuable insights for the advancement of related tofacitinib citrate sustained-release tablet products. Further studies on the stability and bioequivalence of form II are warranted in the future.
